# Supplementary material for: Hyperammonaemia in Dogs Presenting with Acute Epileptic Seizures—More than Portosystemic Shunts
Source: Animals (Basel). 2025 Aug 30;15(17):2558. doi: 10.3390/ani15172558 (PMC12427340; doi:10.3390/ani15172558)
Supplement: Supplementary file 1 [file animals-15-02558-s001.zip › animals-3750817-supplementary.pdf]

Dog	Breed	Age (mo)	Sex	Weig ht (kg)	Type of seizure	Seizure duration/ numbers of seizures	Descripti on of seizures	Level of consciousn ess at presentatio n	Ammonia value at presentati on (RI 14-54 µmol/l)	Ammonia analysed < 8 h after seizure	Ammonia analysed > 8 h < 24 h after seizure	Diagnostics Blood analysis, Urine analysis, CSF analysis	Diagnostic imaging	Diagnosis	Cause of hyperammon aemia	Follow up /Outcome	Additional information
1	Groenen-dael	2	M	3,93	CS	2 CS within 18 h	GTCS	Impaired	112	Within 6 h	X	Haematology, chemistry: ALAT 0,2 µkat/l (<1,2), ALP 5,7 µkat/l (<1,4), Bilirubin 0,3 µmol/l (<2,8), BA-stimulation normal, pre-prandial BA 4 µmol/l (<20), post-prandial BA 9 µmol/l (<25), Hct 32% (41-57), P 2,7 mmol/l (0,7-1,9), TP 45 g/l (49-71), Ca 3,1 mmol/l (2,4 -3), urea 4 mmol/l (3-9), Creatinine 29 µmol/l (<135)<br><br>Urine analysis: WBC 21-50/visual field, occasional coliform bacteria	CT angiography liver normal,<br>no evidence of PSS	Not confirmed	Urease-producing E.coli<br><br>No evidence of PSS No conclusive laboratory findings supporting acute liver failure	Normal NH3 44 µmol/l (14-54) at follow up after 3 weeks	Concurrent UTI with E. coli when admitted for seizures, treated with amoxicillin. Recurrent seizures despite normalized ammonia.<br>3 months later MRI brain; T2 hyperintense lesion in Rt piriform lobe with normal CSF analysis
2	Rottweiler	3	F	12,1	CS	2 CS within 24 h	GTCS	Impaired	73	Within 6 h	X	Haematology, chemistry: ALAT 0,2 µkat/l (<1,2), ALP 1,5 µkat/l (<1,4), BA 3 µmol/l, Post-prandial BA 1 µmol/l (<25), Hct 32% (37-62), Glucose 6,9 mmol/l (3,7-6,6), Urea 3 mmol/l (3-9), Creatinine 28 µmol/l (<135), P 2,6 mmol/l =0,7-1,9)<br><br>Urine analysis: RBC 4-10/visual field, moderate amount of struvite crystals<br>Drug test urine - positive for benzodiazepine (analysed after administration of diazepam to stop seizures)	MRI brain normal<br>Ultrasound abdomen; liver possibly enlarged, normal parenchyma, no signs of PSS	Suspected reactive seizures	No definitive cause identified<br>No evidence of PSS<br>No conclusive laboratory findings supporting acute liver failure	BA 1 µmol/l (<20-25) at follow up after 2 weeks	Intoxication (sertraline) possible aetiology of seizures
3	Chihua-hua Longhair	8	M	1,55	CS	10 CS within 12 h	Focal start progressi ng to GTCS	Impaired	240	X	Within 12 h	At presentation for seizures: haematology, chemistry WNL, ALAT 69 U/L (10-100), ALP 80 U/L (23-212) BA 40 µmol/l (<20-25),<br>Creatinine 57 µmol/l (<135)<br>CSF: WBC 8/µl (6 MN, 2 PM) (<5/µl)	CT angiography liver normal, no signs of PSS<br>MRI brain normal	MUE	No definitive cause identified<br>No evidence of PSS	Slightly elevated NH3 64 µmol/l (14-54), BA 39 µmol/l (<20-25), ALP 1,8 µkat/l (<1,4) after 6 weeks (treated w phenobarbital and prednisolone)<br>Normal bile acids at repeated follow ups for 6,5 years<br>History of low-grade GI symptoms	Treated with phenobarbital and prednisolone for seizures and MUE,<br>follow up bloodwork between November 2014 (presented for CS in October -14) until April 2021:<br>BA 14 µmol/l; 27 µmol/l; 7 µmol/l; 0 µmol/l; 6 µmol/l; 0 µmol/l (<20-25),<br>ALP: 2.3 µkat/l; 3,0 µkat/l; 1,7 µkat/l, 4.4 µkat/l, 9.2 µkat/l, 7,2 µkat/l, 9.3 µkat/l (<1,4)

																months bf seizures	
4	Yorkshire Terrier	13	F	1,7	CS->SE	Unknown number of CS during 8h progressing to SE of at least 10 min duration	GTCS	Impaired (presented during SE)	75	Within 8 h	X	Haematology, chemistry: ALAT 81 U/L (10-125), ALP 70 U/L (23-121), BA 25 µmol/l (<20-25),<br>Hct 34,2% (37,3-61,7), Creatinine 25 µmol/l (44-125), Glucose 3,7 mmol/l (4,11-7,95), TP 50 g/l (52-82), CRP 50 mg/L (<20)	NP	Not confirmed	No definitive cause identified<br>No conclusive laboratory findings supporting acute liver failure	Died during admission, no post-mortem exam	Fracture surgery 48 h prior to seizures
5	Papillon	66	MN	4,46	SE	3 h	GTCS	Impaired	101	X	Within 12 h	Haematology, chemistry: ALAT 0,9 µkat/l (<1,20), ALP 4,4 µkat/l (<1,4), , Bilirubin 2 µmol/l (<3,00), pre-prandial BA 2 µmol/l (<20),<br>WBC 19,2 x10*9/l (4,90-14,70) Urea 6 mmol/l (3-6) , Creatinine 38 µmol/l (<135), Ca 2,0 mmol/l(2,4-3,0), K 2,9 mmol/l (3,4-5,0), Cl 100 mmol/l (106-120, Glucose 2,4mmol/l (3,7-6,6), CRP 120mmol/l (<20 mmol/l)	Exploratory laparotomy: liver of normal size and texture	Hypoglycaemia<br>Systemic inflammation	No definitive cause identified<br>No conclusive laboratory findings supporting acute liver failure	Euthanasia, no post-mortem exam	History of long term IBD, treated with prednisolone and ciclosporin, presented with suspicion of gastrointestinal corp al and hypoglycaemia, continued to seizure after the hypoglycaemia was corrected, then ammonia was analysed
6	Mixed breed	6	F	4,85	CS	4 CS within 12 h	GTCS	Normal	58	X	22 h	Haematology, chemistry: ALAT 0,6 µkat/l (<1,2), ALP 2,6 µkat/l (<1,4), BA 1 µmol/l (<20),<br>Creatinine 52 µmol/l (<135), Urea 7 mmol/l (3-9), P 2,1 mmol/l (0,7-1,9)	NP	Not confirmed	No definitive cause identified<br>No conclusive laboratory findings supporting acute liver failure	Follow up not available	
7	Chihua-hua Longhair	8	M	2,5	CS	2 CS within 1 h	GTCS	Impaired	84	Within 6 h	X	Haematology, chemistry:<br>ALAT 81 U/L (8-75), ALP 74 U/L (23-212), Bilirubin 3 µmol/l, BA not analysed<br>Glucose 8,2 mmol/l (4,2-6,1) (0-15) Creatinine 46 µmol/l (44-159), Urea 4,6 mmol/l (2,5-9,6)	NP	Not confirmed	No definitive cause identified<br>No conclusive laboratory findings supporting acute liver failure	Died during admission, no post-mortem exam	
8	Border Collie	28	F	16,8	CS->SE	CS progress-ed to SE duration not specified	GTCS	Impaired (presented during SE)	106	X	>12 h < 24 h	Haematology, chemistry: ALAT 2,0 µkat/l (<1,2), ALP 1,5 µkat/l (<1,4), Bilirubin 9 µmol/l (<2,8), BA 155 µmol/l (<20-25),<br>Hct 37 % (41-57), Glucose 8,9 mmol/l (3,7-6,6)	Ultrasound: liver assessed as normal	Hepatic encephalopathy / hepatopathy	Suspected re-canalisation or persistent shunting after surgery causing elevated BA and ammonia, alone or in		Surgically corrected PSS as puppy<br>No information about type of PSS (intra-or extrahepatic)<br>Possible persistent shunting or re-canalization causing elevated BA and ammonia<br>despite normal ultrasound, or in combination with motor activity during seizure<br>Sire fathered puppies with IE

															combination with motor activity during seizure and elevated lactate		
9	Miniature Pinscher	4	F	2,5	CS	19 CS within 96 h	Focal motor +GTCS	Impaired (presented during SE)	325	3 h	X	Haematology, chemistry: ALP 236 U/L (23-212), post-prandial BA 156 µmol/l (<25), Hct 29,7% (37,3-61.7), P 2,34 mmol/l (0,81-2,20), Creatinine 20 µmol/l (44-159)	Extra-hepatic PSS detected on ultrasound, confirmed by CT angiography and surgery	Hepatic encephalopathy / hepatopathy	PSS		Inter-ictal intermittent impaired mentation and bizarre behaviour before diagnosis and correction of PSS
10	Miniature Pinscher	164	F	5,45	SS	<5 min	GTCS	Normal	142	2,5 h	X	Haematology, chemistry: ALAT 3,6 µkat/l (<1,2), ALP 9,5 µkat/l (<1,4), BA stimulation performed at ref clinic: abnormal<br>PLT 673 x 10*9/L(108-562), TP 78 g/l (49-71)	NP	Hepatopathy	Hepatopathy		History of elevated liver enzymes and abnormal BA-stimulation. Ultrasound abdomen at referral clinic: abnormal liver

Table S1. Descriptive and diagnostic information of the ten dogs with hyperammonaemia presenting with recent or ongoing seizures.

The biochemistry profiles included either “Normal blood status dog” including CRP, glucose, urea, ALP, ALAT, creatinine, albumin, protein, calcium, phosphate, bile acids, sodium, potassium, chloride or “Chemistry” including bilirubin, CRP, cholesterol, triglycerides, glucose, urea, ALP, ALAT, creatinine, albumin, phosphate, protein, calcium, sodium, potassium, chloride or “Out-of-hours profile” including albumin, ALP, ALAT, urea, creatinine, glucose, protein, sodium, potassium and chloride. The type of profile analysed was depending on the choice of the clinician or the time of day the dog arrived at the hospital. Haematology included as a minimum haematocrit, erythrocytes, total count white blood cells and platelets.

Only the values that either deviated from the reference intervals in the respective haematology or biochemistry profiles or are relevant to conclude if the dog had acute liver failure or PSS or not are included in Table 1. Values not shown in the table should be considered within the reference interval.

F=female, FN=female neutered, M=male, Male neutered, CS=cluster seizures, SE=status epilepticus, GTCS= generalised tonic-clonic seizures, MRI=Magnet Resonance Imaging, CT= Computed Tomography CSF=Cerebrospinal fluid, UTI=Urinary tract infection, PSS=portosystemic shunt, NP=not performed, NA=not analysed WNL=within normal limits, MUE=meningoencephalitis of unknown aetiology, IBD=inflammatory bowel disease, IE=idiopathic epilepsy, PM=post mortem examination, FLAIR= Fluid-attenuated inversion recovery, T2= T2 weighted, AED=antiepileptic drugs
